# Changes in Mouse Gut Microbial Community in Response to the Different Types of Commonly Consumed Meat

**DOI:** 10.3390/microorganisms7030076

**Published:** 2019-03-11

**Authors:** Zhimin Zhang, Dapeng Li, Rong Tang

**Affiliations:** 1 College of Fisheries, Huazhong Agricultural University, Wuhan 430070, China; zhangzm@ihb.ac.cn (Z.Z.); tangrong@mail.hzau.edu.cn (R.T.); 2 Institute of Hydrobiology, Chinese Academy of Sciences, Wuhan 430072, China; 3 Hubei Provincial Engineering Laboratory for Pond Aquaculture, Wuhan 430070, China

**Keywords:** meat consumption, gut microbiota, lipid metabolism, lipopolysaccharide-binding protein (LBP)

## Abstract

The consumption of various meats prevalent throughout the world affects host health probably by associating with compositional shifts of gut microbiota. However, the responses of gut microbiota to different types of meat are not well understood. In this study, we explored the effects of cooked fish (white meat), and pork and beef (red meat) on gut microbiota and blood lipid metabolism in male C57BL/6 mice by comparing to those fed laboratory chow. Significant differences in microbial communities were observed among meat- and chow-fed mice. Compared with the chow group, the red and white meat groups obviously increased in abundance of *Clostridium*, and decreased in *Prevotella* abundance. The richness and diversity of gut microbiota were markedly decreased in the two red meat groups, with lower abundance of *Oscillospira* and higher abundance of *Escherichia*. Meanwhile, there were significant meat-related differences in blood lipid metabolites, with lower levels of high-density lipoprotein, low-density lipoprotein, cholesterol, and in mice fed white, compared with red, meat. Lipopolysaccharide-binding protein was significantly lower in fish-fed mice. Our results indicate that different types of meat potentially influence gut microbial compositions and blood metabolic profiles, suggesting a need to focus on clinically relevant bacteria in gut microbiota associated with increasing meat consumption.

## 1. Introduction

The mammalian gastrointestinal tract is colonized by trillions of microorganisms that influence the health and immune responses of the host. Distinctive compositional patterns of human and other mammalian gut microbiota have been described, driven primarily by diet, lifestyle, and culture [1,2,3]. Altered gut microbiota have been linked to various inflammatory responses [4,5] and diseases [6,7]. Meanwhile, worldwide dietary shifts to refined fats, refined sugar, and meats have contributed to increasing overweight and obesity [8], which has triggered an increase in chronic non-communicable diseases [7]. There is strong evidence that changes in fat, carbohydrate, and fiber in diets have far-reaching effects on gut microbiota [9,10,11] or even cause specific microbiota to become extinct [12].

Recently, a study in mice fed different types of protein reports that meat proteins lead to similar changes in the overall composition of gut bacteria, but different from changes resulting from intake of non-meat proteins [13]. The effects of changes in specific diets were not described in those macronutrient studies. Currently, dietary interventions in humans and animal models are generally designed to investigate microbial community dynamics or responses [14,15,16]. Despite extensive investigations of gut microbiota of human and mammals spanning a range of diet-based controlled factors such as animal- or plant-based dietary intervention [11,15] or dietary styles [1,3], it remains unclear how gut microbiota compositions change in response to dietary supplementation of specific types of meat.

As a vital component of the human diet, the consumption of meat is increasing, and is an important determinant for human health in modern societies. Epidemiological studies have revealed that meat consumption is associated with incidences of various diseases, and that the impacts may be closely associated to the type of meat. Red meat such as pork and beef might contribute to obesity [17], cancers [18], and coronary heart disease [19], whereas white meat (such as fish) may have positive effects [20,21]. Previous in vitro studies have reported the effects of different meats on the composition of human fecal microbiota, indicating that interactions between the type of meat and gut microbiota are correlated with increased risk of intestinal diseases or complications associated with high intake of certain meat [22]. However, it is difficult to relate in vivo gut microbiota compositions with host physiology. Koeth et al. (2013) demonstrated that intestinal microbiota may act to link l-carnitine-rich red meat and atherosclerosis risk in mice [23]. Therefore, there is a need to study the direct responses of gut microbiota to different types of meat in humans or animal models.

In this study, our objective was to mainly investigate the responses of gut microbiota in mice to three different meats for the further knowledge on the link between meats and gut microbiota. We supplied mice with red meat (pork and beef), white meat (fish), and laboratory chow (chow), and provided insight into host blood metabolism associated with the meat consumption. To this end, we also measured blood metabolic indices. In addition, fecal properties such as weight and moisture content were monitored.

## 2. Materials and Methods

### 2.1. Animals and Feeding Schedule

Forty male C57BL/6 mice were, at 3 weeks of age, obtained from the Hubei Research Center of Laboratory Animals, Wuhan, China. They were randomly assigned to four groups of ten each (*n* = 10, five mice/cage). All experiments were approved by and performed in accordance with the guidelines of the Huazhong Agricultural University Animal Care and Use Committee under the approved study number HZAUMO-2016-026.

After the environmental and dietary adaptation for 3 weeks, mice in four groups were selected to be fed chow, cooked fish, pork, and beef, respectively, at 9:00 and 18:00 daily for 8 weeks. All fresh edible meats (fish, pork, and beef) were obtained from a local market and subjected to steamed processing for 15 min before feeding, and the chow was purchased from the Hubei Research Center of Laboratory Animals. All animals were supplied with water available ad libitum. Uneaten food was removed about 2–3 hours after feeding and the weight of the uneaten food was measured. During the experimental period, the body weight of mice was measured. Feces used for the measurements of fecal characteristics were collected at 15:00–17:00 at the fourth week and for gut microbial community analysis at the end of week 8. Additionally, the liver, spleen, and heart were collected and then weighed, and serum samples were obtained at the second day for the analysis of blood lipid metabolites and an inflammation marker, lipopolysaccharide-binding protein (LBP), by centrifugation at 3000× *g* for 20 min. The samples for microbial analysis and serum samples were immediately stored at −80 °C for further analysis.

### 2.2. DNA Extraction and Sequencing

The DNA was extracted from about 150 mg of 40 fecal samples with the QIAamp DNA Stool Mini Kits (Qiagen, Hilden, NRW, Germany) following the manufacturer’s instructions. The V4–V5 hypervariable region amplicons of 16S rRNA genes of the DNA was amplified using the primers: F 515 (5′-GTGCCAGCMGCCGCGG-3′) and R 907 (5′-CCGTCAATTCMTTTRAGTTT-3′) [24]. All PCR assays were carried out with Phusion® High-Fidelity PCR Master Mix (NEB, Ipswich, MA, USA). The resulting amplicons were purified with Qiagen Gel Extraction Kits (Qiagen, Hilden, NRW, Germany) and sequenced with the Illumina HiSeq 2500 platform following standard protocols. The sequences are available at the Sequence Read Archive of the NCBI under accession number PRJNA355989.

### 2.3. Bioinformatics Analysis

Sequence assembly was performed after truncating the paired-end reads to exclude sequence overlaps using FLASH. Default parameters in the QIIME pipeline were set for quality filtering by de-multiplexing the data and removing certain reads using QIIME [25]. Chimera sequences were discarded by comparing the obtained tags with the reference database (Gold database, http://drive5.com/uchime/uchime_download.html) using the UCHIME algorithm. Operational taxonomic units (OTUs) were defined using a 97% threshold of similarity by UPARSE [26] and each sequence was assigned a taxonomy using the Ribosomal Database Project [27] and the Greengenes database [28], followed by phylogenetic relationship construction using MUSCLE [29]. Rarified OTU tables with a standard number of sequences corresponding to the sample with the least sequences were generated. The alpha and beta diversities were calculated with QIIME based on the normalized OTUs abundance information. An OTU-based unweighted UniFrac distance matrix was calculated in QIIME and visualized using the first two coordinates in a principal coordinate analysis (PCoA).

### 2.4. Blood and Fecal Assays

One serum sample was not available in the beef-fed group. Serum lipopolysaccharide-binding protein (LBP) was assayed with a commercial ELISA Kit (Boster Biological Technology Co., TLD, Wuhan, China) following the manufacturer’s instructions. Low-density lipoprotein (LDL), high-density lipoprotein (HDL), total cholesterol (TC), triglycerides (TG), blood urea nitrogen (BUN), alkaline phosphatase (ALP), albumen (ALB), glutamic-oxalacetic transaminase (AST), glutamic-pyruvic transaminase (ALT), total protein (TP), and glucose (GLU) in serum were determined using a CHEMIX-800 autoanalyzer (Sysmex Infosystems, Kobe, Japan) and commercially available kits (Nanjin Longkui Biological Technology Co., Ltd, Nanjin, China) following the manufacturer’s protocols. The weight and moisture content of fresh feces were evaluated in consecutive 4-day fecal collections at fourth week.

### 2.5. Diversity and Statistical Analysis

Differences in alpha diversity, blood parameters, fecal weight, and fecal water content among groups were tested by non-parametric, one-way analysis of variance (ANOVA) and the Kruskal–Wallis test in SPSS 20.0 (SPSS Inc., Chicago, IL, USA). Unweighted UniFrac distances were ordinated by PCoA. Analysis of similarities (ANOSIM) was used to test the significance of differences in microbial community distances within the matrix constructed from the microbial sequences.

## 3. Results

### 3.1. Taxonomic Structure and Characterization

**Figure 1:**
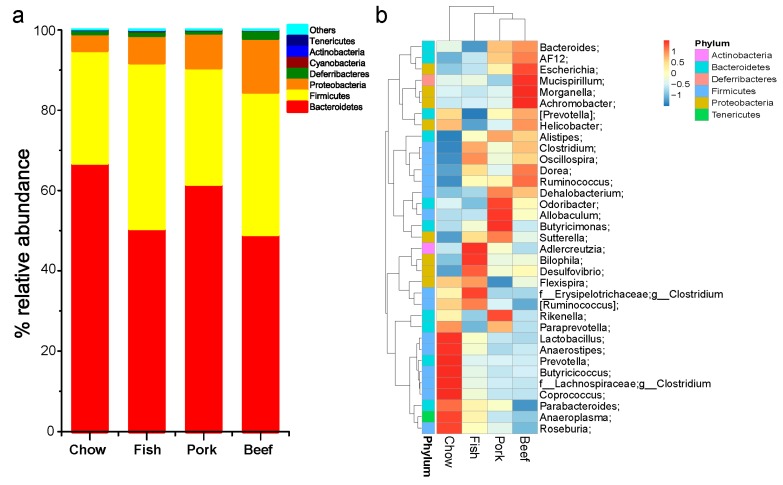
Taxonomic compositions of gut microbiota in mice fed laboratory chow, fish, pork, or beef. (**a**) Relative taxa abundance of gut microbiota of mice fed chow, fish, pork, or beef, summarized at the phylum level; (**b**) heatmap for cluster analysis of gut microbiota for the 35 most abundant genera in the four groups.

Bacterial sequences were obtained from fecal samples used in this study (Table S1). Taxonomically, seven different bacterial phyla were identified, with three phyla *Bacteroidetes*, *Firmicutes*, and *Proteobacteria* dominating in the gut. Within each group, there were some individual variations of gut bacterial compositions (Figure S1). The bacterial communities were dominated by three phyla, the most abundant was *Bacteroidetes*, with 48.15% to 83.11% of the sequences, followed by *Firmicutes* with 14.05% to 42.41%, and *Proteobacteria* with 2.19% to 7.85% in the chow-fed group. The overall relative abundance in the three meat-fed groups was 37.33% to 76.99% for *Bacteroidetes*, 16.86% to 53.43% for *Firmicutes*, and 4.38% to 21.56% for *Proteobacteria* (Figure S1). A decreased abundance of *Bacteroidetes* was observed in the three meat-fed groups compared with the chow-fed group, while the abundance of *Proteobacteria* increased in all meat-fed mice; *Firmicutes* increased in the fish- and beef-fed groups, but not in the pork-fed group (Figure 1a).

**Figure 2:**
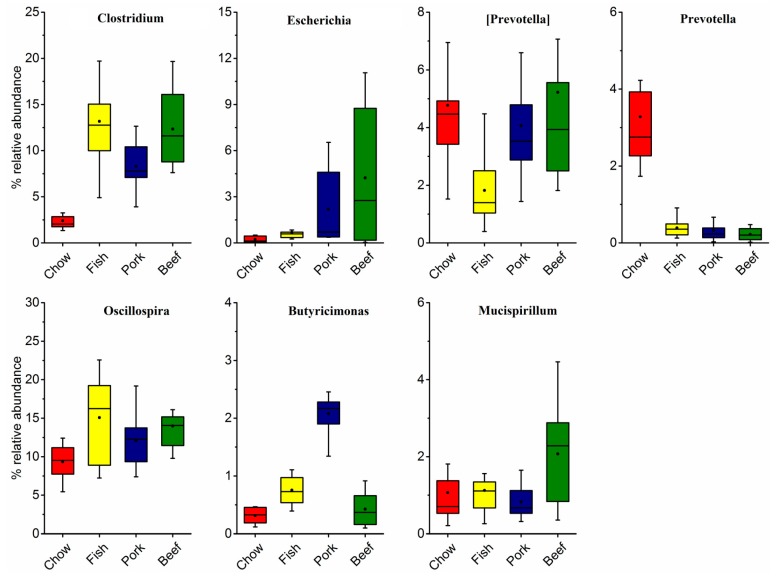
Relative abundance of the most abundant genera in the gut microbiota of mice fed laboratory chow, fish, pork, or beef.

At the family levels, *S24-7* and *Ruminococcaceae* were the two largest proportions of taxa, accounting for 44.65% and 10.79% of gut microbiota in the chow-fed group. The relative proportion of family S24-7 decreased by 32.30% in mice fed with fish, 36.13% with pork, and 28.06% with beef. Family *Ruminococcaceae* increased by 16.88% with fish, 13.91% with pork, and 16.21% with beef. Further characterization of taxonomic compositions revealed microbial changes (Figure 1b and Figure 2). The increased abundance of genera *Oscillospira* and *Clostridium* were largely responsible for the increase in phylum Firmicutes in mice fed with meat. The pork-fed group had the lowest abundance of genus *Prevotella*, and that was significantly higher than in chow-fed mice. Genus *Escherichia* was significantly enriched in all the meat-fed groups, with the highest level in the beef-fed group and the lowest in the fish-fed group (Figure 2). 

### 3.2. Meat Consumption Reduces Richness and Diversity of Gut Microbiota

**Figure 3:**
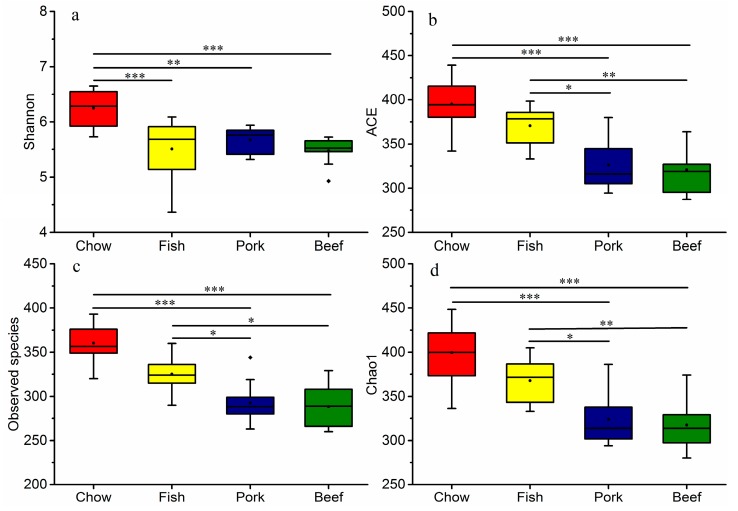
Differences in gut microbial diversity and richness in mice fed laboratory chow, fish, pork, or beef. Whiskers in the boxplot represent the range of minimum and maximum alpha diversity values within a diet group. The black dot inside or outside the boxplot are means and outliers, respectively. The horizontal line in the boxplot is the median. * *p* < 0.05, ** *p* < 0.01, *** *p* < 0.001. (**a**) Shannon diversity; (**b**) ACE; (**c**) Observed species; (**d**) Chao1.

Several different metrics were used to measure alpha diversity, including Shannon diversity index, ACE, Chao1, and observed species. Similar diversity estimates (Shannon index, Figure 3a) were observed in mice fed with different types of meat (*p* > 0.05), but it was significantly lower than that in mice fed chow (*p* < 0.05). Meat-fed groups had a lower microbial richness than chow-fed group (*p* < 0.05), and the trend was observed in both observed and estimated richness metrics (Chao1 and ACE, Figure 3c,d). Richness in the white-meat-fed (fish) group was consistently higher than the red-meat-fed (pork and beef) groups (*p* < 0.05). However, the diversity and richness of the two red-meat-fed groups were not significantly different (*p* > 0.05) (Figure 3).

### 3.3. Multivariate Analysis of Gut Microbial Responses to Meat

Principal coordinate analysis distinguished mice fed red meat from those fed white meat, and from mice fed chow (Figure S2). Furthermore, the clustering of gut microbiota at the genus levels clearly revealed the separations of mice fed red meat, white meat, or chow, indicating different effects of meat types on gut microbial composition (Figure 1b). ANOSIM confirmed the significant differences in microbial community in the different experimental groups. The data are summarized in Table S2. The *R*-value for the two red-meat-fed groups (*R* = 0.32, *p* = 0.002) was lower than that for the pork- and fish-fed groups (*R* = 0.36, *p* = 0.002), and the beef- and fish-fed groups (*R* = 0.59, *p* = 0.001).

### 3.4. Growth, Blood Metabolic Indices, and Fecal Characteristics

**Figure 4:**
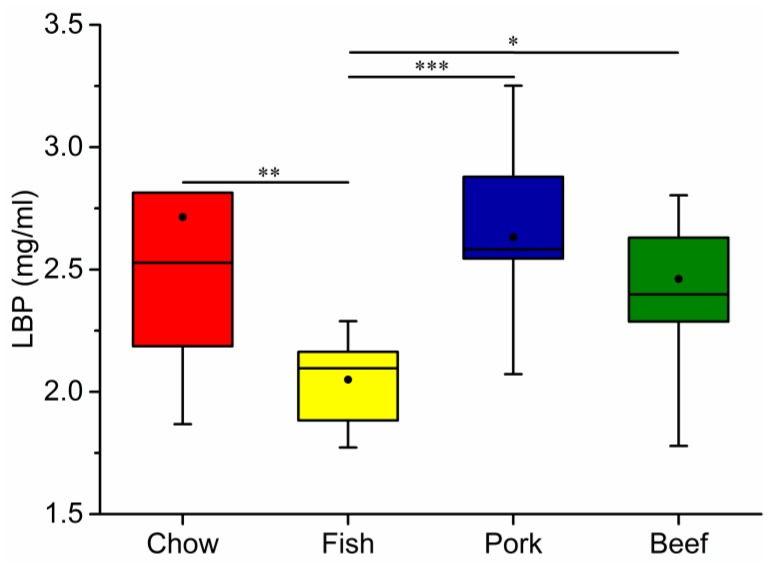
Changes in serum lipopolysaccharide-binding protein (LBP) levels of mice fed laboratory chow, fish, pork, or beef. The numbers of mice in each group were: chow, 10; fish, 10; pork, 10; and beef, 9. Whiskers in the boxplot represent the range of minimum and maximum alpha diversity values within a diet group. The black dot in the boxplot is the mean. The horizontal line in the boxplot is the median. * *p* < 0.05, ** *p* < 0.01, *** *p* < 0.001.

**Figure 5:**
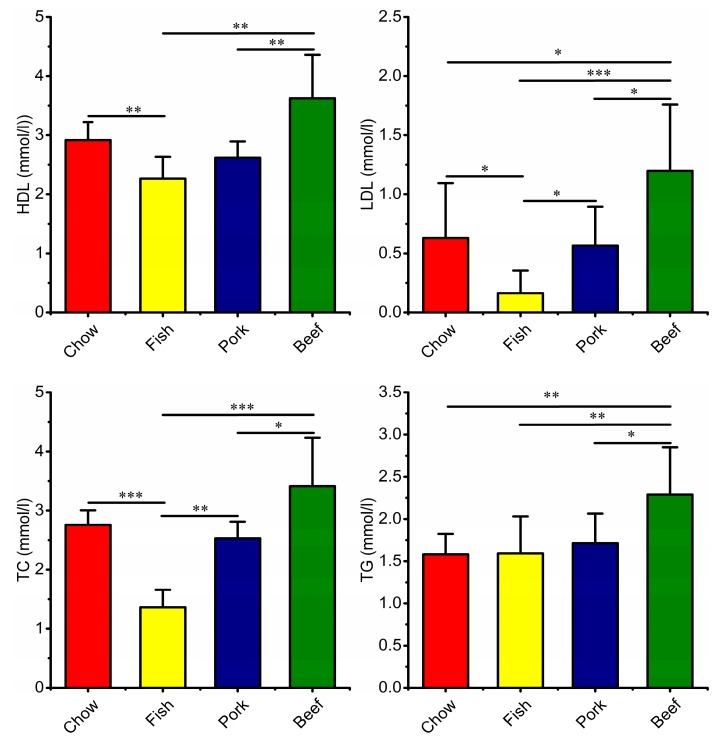
Differences in blood metabolites of mice fed laboratory chow, fish, pork, or beef. HDL, high-density lipoprotein; LDL, low-density lipoprotein; TC, total cholesterol; TG, triglycerides. Data are mean ± SD, the numbers of mice in each group were: chow 10; fish, 10, pork, 10; beef, 9. * *p* < 0.05, ** *p* < 0.01, *** *p* < 0.001.

The results showed that the chow-fed group gained more body weight than the meat-fed groups at the end of week 8. There were no significant differences in growth, including body weight and the weight of internal organs among the three meat-fed groups (Figure S3). The fish-fed group had the lowest level of LBP in the four groups (Figure 4), with a significantly elevated level of LBP in red meat-fed groups. Meanwhile, fish-fed group had the lowest levels of serum HDL, LDL, TC and TG, all of which were significantly increased in red-meat-fed groups, with higher values in the beef-fed group (Figure 5). Blood urea nitrogen was significantly higher in the meat-fed mice than chow-fed mice. Conversely, the chow-fed group had the highest GLU level (Table S3). In addition, fecal particles were larger in the chow-fed than in the three meat-fed groups (Figure S4a). The feces of the red-meat-fed groups contained lower moisture contents compared to that in fish- or chow-fed groups (Figure S4b).

## 4. Discussion

In this study C57BL/6 mice were used to identify the responses of gut microbiota to dietary meat supplements and the changes in microbial compositions were significantly observed. Although fecal microbiota analyzed in this study does not completely reflect the communities of the digestive track, they are representative for communities and commonly used in humans and animals [14,15,16,30]. The microbial profiles associated with meat consumption were distinct from those in chow-fed animals. Some specific shifts in microbial composition appeared to be responses to different diet characteristics, which parallels the findings of previous studies focusing on alterations in the compositions of gut microbiota caused by macronutrient manipulation [8,11,12]. The results further provide preliminary evidence of meat-specific associations with gut microbiota that ultimately may affect host health.

It is widely accepted that diet and eating habits can significantly modulate the gut microbiota, despite the existing profiles depending on a series of host and environmental factors [2,3,9,14,31]. In this study, we found the microbial community structure of red-meat-fed mice was more similar to each other than that of fish-fed mice. The analogous microbial richness was seen in mice fed with red meat, but red meat consumption profoundly reduced the diversity of gut microbiota compared with fish consumption. The phylogenetic differences of gut microbiota associated with meat consumption may reflect specific, but not overall changes in taxa, associated with dietary components [12]. Low richness of gut microbiota has been reported in obese individuals with high-protein and high-fat western diets [1], and in elderly patients with inflammation [32]. However, differences in richness and diversity associated with intake of specific meat have not previously been reported. It is possible that increased microbial richness associated with fish consumption may have health benefits. In addition, the decreased moisture content of fecal materials may limit microbial growth by reducing nutrient mobility and enzymatic activity [33], and, as shown in this study, the moisture content of feces is negatively correlated with species richness [34,35]. Thus, high levels of red meat intake may decrease the stability of the gut microbial ecosystem [36] and the adaptive potential of gut microbiota [1].

A series of intestinal bacteria are involved in the fermentations of food consumed [37]. Some *Clostridium* spp. are common intestinal bacteria which degrade proteins and amino acids, such as protein-rich meat. It has been reported that *Clostridium perfringens* is associated with proteolytic activity [38]. The advantage of *Clostridium* being mostly dominated by *C. perfringens* in meat-fed mice from this study potentially indicates the functional role of protein utilizations. The rapid increase of *C. perfringens* was also found in a previous in vitro study of human fecal microbiota cultured with various kinds of meat [22]. However, the abundant *C. perfringens* can also secrete high levels of enterotoxins including amines, indoles, and ammonia, generally leading to intestinal pathologies [6,39,40].

A typical western diet can significantly alter gut microbiota. For example, it can result in an increased abundance of *Escherichia coli* in humans [1]. This result is consistent with our observation that mice fed red meat had higher abundance of *Escherichia* (mostly belonging to *E. coli*), but not in mice fed fish. This means that meat consumption will expose animals to *E. coli* through different contributions of meat types. In humans, it also exists. In the Dutch population, chicken meat accounted for the majority of total *E. coli* load on meat, but a relatively larger effect of heating on chicken meat than beef was observed during food preparation [41]. Moreover, this taxon may preferentially utilize different amino acids [37]. To some degree, these may explain differences in the abundance of *E. coli* in mice fed red meat and white meat. This phenomenon is not necessarily also for humans, but it deserves more attentions. Compared to meats rich in protein, chow is a commercial feed with high fiber content, which can reduce the *E. coli* count [42] and exhibit a positive correlation of abundant *Prevotella* [15]. Animal-based diets can lead to a prominent increase in *Oscillospira* abundance [15]. We further found more enriched *Oscillospira* in fish-fed mice. Nutritional interventions and human microbiota studies have demonstrated positive associations of *Oscillospira*, leanness, and health [15,43,44], meanwhile *Oscillospira* are bile-resistant and are speculated to utilize mammalian-derived glycans originating either from a diet rich in animal glycoproteins or from the host [45].

The blood assays performed in this study included biomarkers of chronic inflammation or disease onset. LBP reflects the antigen load of the blood and indicates the presence of low-grade inflammation because of its ability to bind lipopolysaccharide. Lipopolysaccharides present in the outer membrane of Gram-negative bacteria produce metabolic endotoxemia when entering circulation because of increased intestinal permeability. Fish intake decreased serum LBP in contrast to a recent study that showed fish protein was associated with higher LBP levels than red meat protein [13]. The difference may have resulted from different dietary components. For example, fish is enriched in long-chain polyunsaturated fatty acids (PUFAs) that can inhibit inflammation and related metabolic disorders [46]. Mujico et al. (2013) reported that n-3 fatty acid supplementation counteracted gut dysbiosis induced in obese mice by a high-fat diet and increased the abundance of *Firmicutes* bacteria [47]. Conversely, red meat rich in fat or proinflammatory cytokines can lead to increased inflammation [23]. In this study there was an unexpected lack of reads assigned to *Lactobacillus* and *Bifidobacterium* both benefiting the host by alleviating inflammation and the metabolic syndrome. Differently, the two taxa were highly abundant in the study of dietary protein in Sprague–Dawley rats [13]. In addition to stimulating the growth of potentially pathogenic species, it was found that excessive protein intake can reduce fecal counts of beneficial *Bifidobacteria* [48]. Therefore, dietary composition and host sensitivity should both be considered.

High HDL levels are associated with a decreased risk of coronary heart disease [49,50]. An increase in HDL in response to red meat consumption clearly contradicts evidence that red meat consumption may be detrimental to health [21]. However, a recent study indicates that HDL may not be causally protective against coronary heart disease [51]. Nevertheless, fish intake is significantly effective at maintaining lower levels of LDL [52], which corresponded to decreased levels of cholesterol and triglycerides, suggesting that fish consumption may be more beneficial for decreased metabolic loadings and for inflammatory mediators due to n-3 PUFA in fish [53].

## 5. Conclusions

The changes in mouse gut microbiota and blood metabolic indexes have been linked to meat consumption. However, white meat (fish) and red meat (pork and beef) significantly resulted in differential responses in gut microbiota and metabolism. Our findings provide an insight that fish may have more beneficial gut microbiota profiles compared with red meat. The significant changes in blood metabolic markers warrant additional studies of meat consumption, especially fish. Targeted metabolomics evaluations are needed to test the hypotheses of how meat consumption changes gut microbiota and how those changes correlate with host physiology. The results will provide a comprehensive understanding of meat-associated gut microbiota composition and diet-specific interventions.

## Supplementary Materials

The following are available online at https://www.mdpi.com/2076-2607/7/3/76/s1, Figure S1: Individual heterogeneity in gut microbiota profiles within mice fed laboratory chow, fish, pork or beef, Figure S2: Principal coordinates analysis (PCoA) of gut microbiota based on unweighted UniFrac distance matrices, Figure S3: Body weight and internal organ weight of mice fed laboratory chow, fish, pork or beef, Figure S4: The fecal characteristics of mice fed laboratory chow, fish, pork or beef, Table S1. Sequence information of fecal samples used in this study. Table S2: ANOSIM of unweighted UniFrac distance against gut microbiota of mice fed laboratory chow, fish, pork or beef diet at OUT levels, Table S3. Blood metabolic indices of mice fed laboratory chow, fish, pork or beef, Table S2: Blood metabolites index of mice fed laboratory chow, fish, pork or beef.
