# Supplementary material for: Changes in Mouse Gut Microbial Community in Response to the Different Types of Commonly Consumed Meat
Source: Microorganisms. 2019 Mar 11;7(3):76. doi: 10.3390/microorganisms7030076 (PMC6462912; doi:10.3390/microorganisms7030076)
Supplement: Supplementary file 1 [file microorganisms-07-00076-s001.pdf]

Supplementary Material

Changes in mouse gut microbial community in response to the different types of commonly consumed meat

Zhimin Zhang[1,2], Dapeng Li[1,3*] and Rong Tang[1,3]

[1]College of Fisheries, Huazhong Agricultural University, Wuhan, 430070, China
[2]Institute of Hydrobiology, Chinese Academy of Sciences, Wuhan 430072, China
[3]HIubei Provincial Engineering Laboratory for Pond Aquaculture, Wuhan, 430070, China
*Correspondence: ldp@mail.hzau.edu.cn; Tel.: +86-27-15397118600 (D.L.)

Supplementary Tables

Table S1. Sequence information of fecal samples used in this study.

Sample ID	Raw PE	Combined	Qualified	Q20	Q30	GC%	Effective%
Chow1	62,503	57,779	55,280	98.55	96.98	53.61	83.99
Chow2	52,433	48,905	46,788	98.6	97.1	53.6	83.67
Chow3	50,091	46,419	44,269	98.52	96.93	54.27	83.89
Chow4	53,645	48,683	46,551	98.53	96.95	53.91	82.16
Chow5	55,827	51,520	49,247	98.54	96.93	54.31	85.12
Chow6	59,437	53,814	51,707	98.54	96.96	53.58	82.39
Chow7	67,452	60,926	58,500	98.56	97	53.59	81.65
Chow8	60,198	54,644	52,572	98.61	97.17	53.41	81.43
Chow9	67,306	61,336	58,965	98.64	97.23	53.78	81.92
Chow10	62,406	56,875	54,677	98.59	97.08	53.68	81.81
Fish1	50,460	45,548	43,786	98.62	97.21	53.2	81.33
Fish2	54,605	50,279	48,296	98.68	97.24	53.59	82.91
Fish3	59,811	54,918	52,671	98.62	97.18	53.09	83.39
Fish4	60,828	55,598	53,262	98.6	97.14	52.99	80.83
Fish5	65,207	59,839	57,290	98.59	97.14	53.39	83.05
Fish6	64,807	58,671	56,261	98.54	97.01	53.13	82.3
Fish7	62,720	57,173	54,760	98.6	97.11	53.46	82.29
Fish8	50,567	46,212	44,391	98.63	97.19	53.46	83.61
Fish9	68,430	62,067	59,533	98.59	97.08	53.59	83.61
Fish10	59,824	54,588	52,441	98.63	97.22	53.07	81.45
Beef1	62,670	56,905	54,774	98.61	97.14	53.24	79.23
Beef2	53,217	48,392	46,511	98.62	97.22	53.11	78.07
Beef3	56,291	51,565	49,627	98.67	97.28	53.51	80.54
Beef4	53,383	48,824	46,995	98.65	97.21	53.53	80.77
Beef5	59,770	54,377	52,439	98.66	97.26	53.58	79.38
Beef6	67,799	61,896	59,658	98.65	97.25	52.83	78.27
Beef7	51,176	47,015	45,189	98.63	97.19	52.97	79.75
Beef8	55,285	50,843	48,939	98.67	97.25	53.29	80.18
Beef9	50,263	46,012	44,198	98.63	97.2	53.64	83.04
Beef10	49,121	44,685	42,925	98.62	97.19	53.14	78.29
Pork1	56,810	52,223	50,215	98.68	97.3	53.25	82.81
Pork2	55,096	50,164	48,373	98.68	97.29	53.18	82.15

Pork3	53,050	48,032	46,161	98.63	97.23	53.03	81.28
Pork4	61,100	55,735	53,703	98.63	97.21	52.78	81.08
Pork5	55,044	50,546	48,482	98.61	97.15	53.27	80.69
Pork6	51,010	46,852	44,864	98.6	97.13	53.24	79.62
Pork7	61,378	56,201	53,744	98.59	97.11	53.44	80.81
Pork8	50,214	45,525	43,632	98.59	97.12	53.46	79.7
Pork9	63,954	58,312	55,927	98.6	97.09	54.02	81.75
Pork10	51,079	46,707	44,938	98.61	97.1	53.78	81.39

Table S2. ANOSIM of unweighted UniFrac distance against gut microbiota of mice fed laboratory chow, fish, pork or beef diet at OUT levels.

Groups	R-value	*P*-value
Fish-Beef	0.5919	0.001
Pork-Beef	0.3186	0.002
Pork-Fish	0.3632	0.002
Chow-Beef	0.9913	0.001
Chow-Fish	0.9504	0.001
Chow-Pork	0.9224	0.001

Table S3. Blood metabolic indices of mice fed laboratory chow, fish, pork or beef.

Blood indices	Chow	Fish	Pork	Beef	*P*-Value
ALB (g/l)	11.66 ± 2.55	10.78 ± 2.19	11.00 ± 1.80	11.73 ± 2.20	ns
ALP (U/l)	154.72 ± 25.62	159.72 ± 28.97	186.34 ± 25.87	171.11 ± 26.70	ns
ALT (U/l)	397.54 ±115.18	759.66 ± 503.68	303.82 ± 171.91	420.20 ± 465.35	ns
AST (U/l)	503.58 ± 147.39	764.94 ± 404.50	401.72 ± 184.90	460.29 ± 410.40	ns
BUN (mmol/)	6.64 ± 3.47a	13.40 ± 3.90b	19.15 ± 2.76c	22.81 ± 4.03c	***
GLU (mmol/l)	11.46 ± 1.33a	8.62 ± 0.89b	9.19 ± 1.50b	8.55 ± 1.48b	**
TP (g/l)	43.56 ± 5.47^{a}	45.76 ± 8.97^{a}	47.52 ± 8.24^{a}	59.62 ± 7.00^{b}	**

ns: no significance, $^{*}P< 0.05$, $^{**}P< 0.01$, $^{***}P< 0.001$.

Supplementary Figures

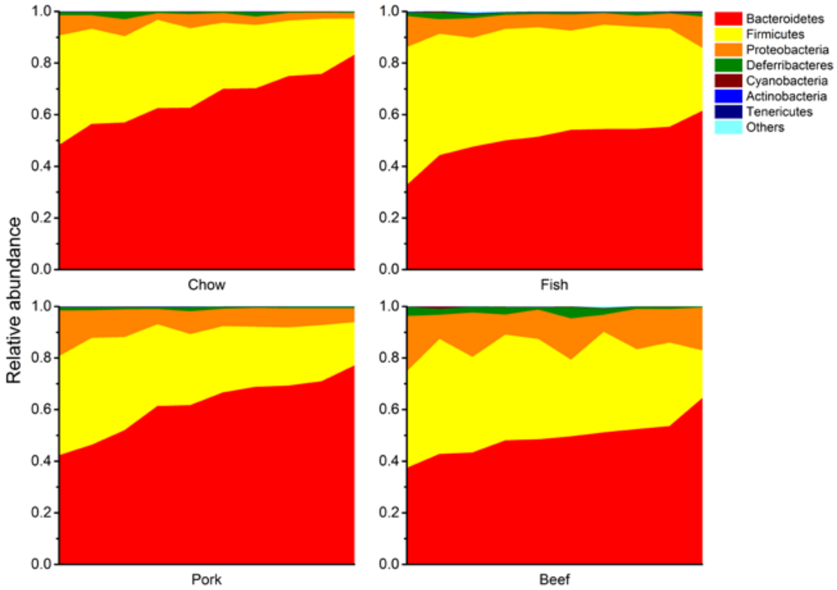

Figure S1. Individual heterogeneity in gut microbiota profiles within mice fed laboratory chow, fish, pork or beef.

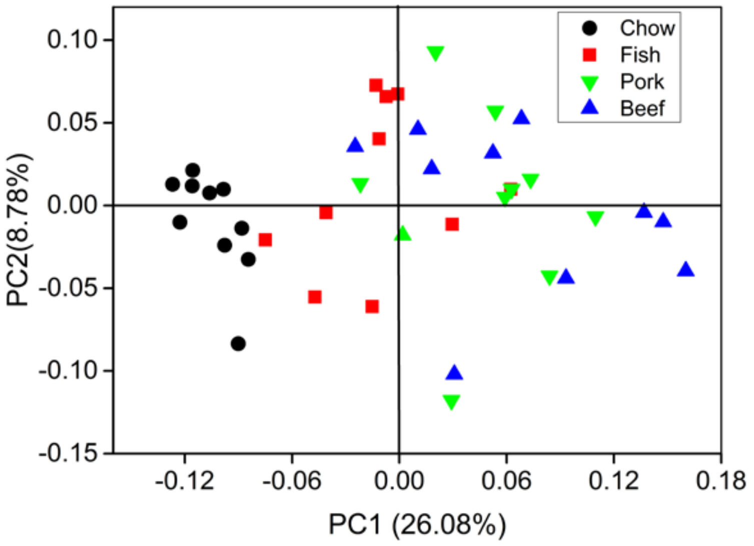

Figure S2. Principal coordinates analysis (PCoA) of gut microbiota based on unweighted UniFrac distance matrices. Mice were fed laboratory chow, fish, pork or beef.

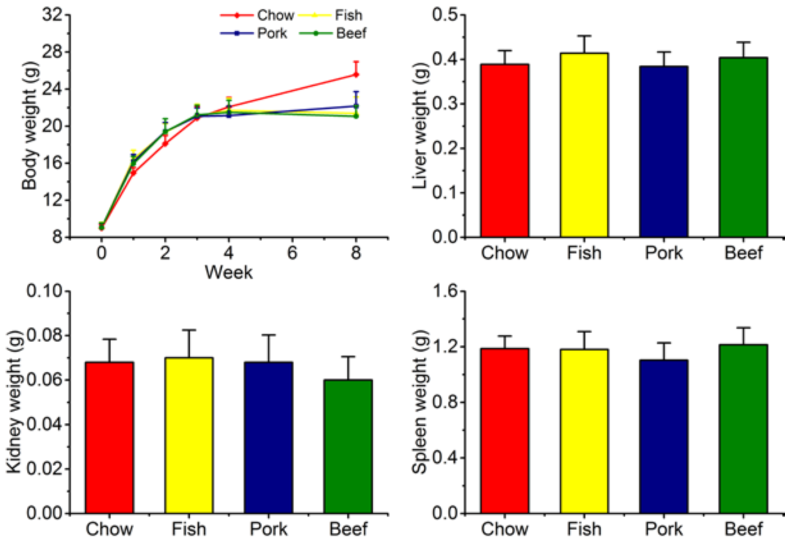

Figure S3. Body weight and internal organ weight of mice fed laboratory chow, fish, pork or beef.

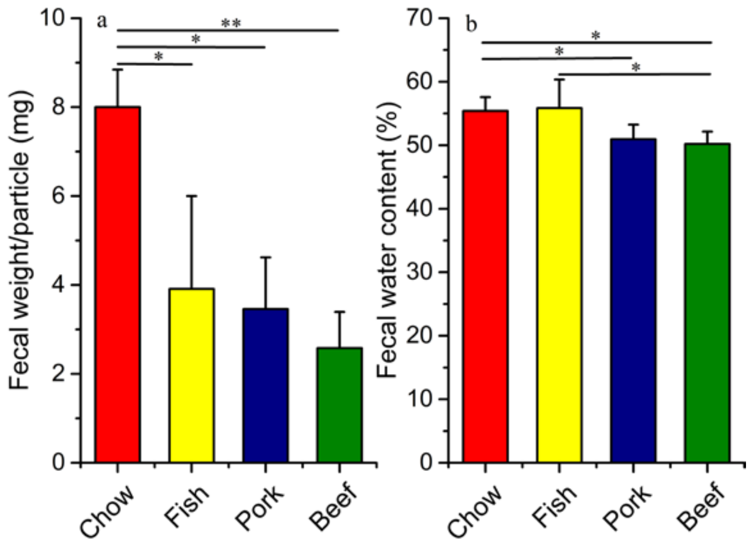

Figure S4. The fecal characteristics of mice fed laboratory chow, fish, pork or beef. (a) The fecal weight per particle; (b) The fecal water content. Data are presented as mean ± SD (standard deviation). $^{*}P< 0.05$, $^{**}P< 0.01$, $^{***}P< 0.001$.
